# Identification of Natural Lead Compounds against Hemagglutinin-Esterase Surface Glycoprotein in Human Coronaviruses Investigated via MD Simulation, Principal Component Analysis, Cross-Correlation, H-Bond Plot and MMGBSA

**DOI:** 10.3390/biomedicines11030793

**Published:** 2023-03-06

**Authors:** Iqra Ali, Muhammad Asif Rasheed, Simona Cavalu, Kashif Rahim, Sana Ijaz, Galal Yahya, Lucky Poh Wah Goh, Mihaela Simona Popoviciu

**Affiliations:** 1 Department of Biosciences, COMSATS (Commission on Science and Technology for Sustainable Development in the South) University Islamabad, Sahiwal Campus, Sahiwal 57000, Pakistan; 2 Department of Biosciences, COMSATS (Commission on Science and Technology for Sustainable Development in the South) University Islamabad, Islamabad Campus, Islamabad 45550, Pakistan; 3 Faculty of Medicine and Pharmacy, University of Oradea, P-ta 1 Decembrie 10, 410087 Oradea, Romania; 4 Department of Microbiology, Cholistan University of Veterinary and Animal Sciences (CUVAS), Bahawalpur 63100, Pakistan; 5 Department of Microbiology and Immunology, Faculty of Pharmacy, Zagazig University, Zagazig 44519, Egypt; 6 Faculty of Science and Natural Resources, Universiti Malaysia Sabah, Jalan UMS, Kota Kinabalu 88400, Sabah, Malaysia

**Keywords:** hemagglutinin esterase, human coronaviruses, lead compounds, molecular docking, pharmacophore model, MD simulation, principal component analysis, dynamic cross correlation, energy decomposition, MMGBSA

## Abstract

The pandemic outbreak of human coronavirus is a global health concern that affects people of all ages and genders, but there is currently still no effective, approved and potential drug against human coronavirus, as many other coronavirus vaccines have serious side effects while the development of small antiviral inhibitors has gained tremendous attention. For this research, HE was used as a therapeutic target, as the spike protein displays a high binding affinity for both host ACE2 and viral HE glycoprotein. Molecular docking, pharmacophore modelling and virtual screening of 38,000 natural compounds were employed to find out the best natural inhibitor against human coronaviruses with more efficiency and fewer side effects and further evaluated via MD simulation, PCA, DCCR and MMGBSA. The lead compound ‘Calceolarioside B’ was identified on the basis of pharmacophoric features which depict favorable binding (ΔGbind −37.6799 kcal/mol) with the HE(5N11) receptor that describes positive correlation movements in active site residues with better stability, a robust H-bond network, compactness and reliable ADMET properties. The *Fraxinus sieboldiana Blume* plant containing the Calceolarioside B compound could be used as a potential inhibitor that shows a higher efficacy and potency with fewer side effects. This research work will aid investigators in the testing and identification of chemicals that are effective and useful against human coronavirus.

## 1. Introduction

Human coronavirus (COVID-19) is a positive, single-stranded RNA virus that causes severe acute respiratory syndrome. It originated in China in 2019 and has spread to more than 210 countries [1]. The coronavirus outbreak appeared to be extremely dangerous and lethal, with approximately 590 million people infected, and over 6.4 million deaths globally [2,3]. The country with the greatest number of patients (around 92.8 million) is the United States of America [4]. Coronaviruses belong to the family *Coronaviridae*, which is further subdivided into four genera i.e., alpha, beta, gamma and delta-corona [5]. Among all of them, β-coronavirus causes more severe disease than other subtypes and according to the phylogenetic analysis of its genome, it shares 82% sequence similarity with SARS-CoV and 50% with MERS-CoV. Novel coronaviruses are lipid-enveloped viruses that are more virulent, pathogenic and contagious [6]. They contain the lengthiest known genome among RNA viruses, with a diameter of 80 to 160 nm [7]. They spread from animals to humans, followed by human-to-human transmission, and morbidity and mortality rates are higher among elderly patients [8,9].

Coronaviruses genomes comprised upon 6 to 10 open reading frames (ORF). ORF1 at 5′ terminal is directly translated into ORF1a and ORF1b, which encode nonstructural proteins, while the remaining ORFs encode some structural and accessory proteins [10]. To understand the virus’s lifecycle, we need to understand the mechanism of these proteins. Therefore, we can determine which protein can be used as a therapeutic target. The nonstructural proteins play a key role in the pathogenesis and survival of the virus inside the cell, while four structural proteins: spike (S), membrane (M), envelope (E) and nucleocapsid (N) proteins, play a significant role in viral replication, attachment [11] and promoting entry into the host cell [12]. The spike protein is important to block viral entry into host cells and thus prevent the virus’s replication [13]. Trimeric S protein is the largest among the other proteins, with a mass of 600 kDa, comprising S1 and S2 subunits [14]. The N-terminal S1 subunit forms the receptor-binding domain (RBD), which helps in the binding of the S protein to the host cell protein angiotensin-converting enzyme 2 (ACE2), while the C-terminal S2 subunit contains fusion machinery and undergoes structural rearrangements during the fusion of viral and cellular membranes [15]. Hemagglutinin-esterase (HE), a viral envelope glycoprotein of approximately 65 kDa, binds to O-acetylated sialic acid of the host cell membrane [16] and aids in the attachment of human coronaviruses [17]. Therefore, the structural spike protein, along with HE, binds to ACE2, which is expressed on the surface of epithelial cells of the lungs, intestines, kidney and blood vessels. Smokers have higher levels of risk of COVID-19 infection than non-smokers because their bodies express the ACE2 gene more than average [18]. After making a connection, HE produces messenger RNA and performs replication [19,20]. This is the reason that the structural protein HE is a potential therapeutic target to inhibit the viral replication.

Many antiviral medications derived from fungi and plants that overcome side effects and increase efficiency were discovered through in vitro tests and computational research for the novel human coronavirus [21]. Polyketides, polyphenols and flavonoids play a significant role against coronaviruses [22]. Plants are viewed as bio-factories due to their antiviral properties and their ability to produce a wide range of chemical compounds with potential medical applications [23]. To combat the coronaviruses, phytochemicals have been investigated for their ability to inhibit the HE protein and prevent coronavirus attachment and replication processes. The use of natural compounds is becoming more effective and gaining importance against viral infections [24].

The objective of this study is to identify natural lead compounds that inhibit the HE surface glycoprotein (5N11). Evaluation of novel inhibitors from library of natural compounds was performed under computational analysis by adopting computer-aided drug design approach with different bioinformatics tools and techniques [25,26,27,28,29]. Ligand’s natural compound’s library available in the SelleckChem database have been screened and identify the lead compound based on its interaction, RMSD, better binding affinity, pharmacophore fit score, and some other parameters [19].

Calceolarioside B was selected as the lead compound due to its ability to inhibit the target protein and it may be used in drug design in the future. Calceolarioside B is a flavonoid glycoside found in the plant *Fraxinus sieboldiana Blume* and has been previously reported for its anti-inflammatory and anti-tumor properties [24]. The results of this study suggest that Calceolarioside B may also have antiviral properties and could be used in the development of novel therapeutics for COVID-19.

## 2. Materials and Methods

### 2.1. Structure Retrieval, Refinement and Evaluation

The X-ray crystallographic structure of Hemagglutinin-esterase (HE) surface glycoprotein with PDB ID 5N11 was retrieved from Protein Data Bank (PDB) (https://www.rcsb.org/ (accessed on 06 March 2022)), a freely available online database that contains the three-dimensional structural data of macromolecules. The structure of the target protein was refined via BIOVIA Discovery studio [30] and evaluated via PROCHECK, which provides information about the stereochemistry of the protein structure via a Ramachandran Plot that describes the quality of the protein [31].

### 2.2. Selection of Ligands and Pharmacophore Generation

Fifteen antiviral ligands were retrieved against the target protein HE surface glycoprotein from the publicly accessible PubChem (https://pubchem.ncbi.nlm.nih.gov/ (accessed on 23 March 2022)) database [32] to generate a pharmacophore query. The use of pharmacophore is effective in computer-aided drug design (CADD). The pharmacophore model is an accumulation of common steric and electronic features that quickly filter through a huge number of a compound’s library for a specific target to initiate or inhibit its biological response. The selected compounds were aligned and analyzed in terms of their chemical characteristics and common features were observed among them, such as hydrogen bond donor, acceptor, cationic, anionic, aromatic and hydrophobic [33]. Protein–ligand interactions were interpreted to achieve steric features. The pharmacophore of 15 active antiviral inhibitors was generated on the basis of RMSD values, common steric and chemical features, and a high binding affinity against Hemagglutinin esterase glycoprotein of human coronavirus using Ligand Scout 4.1.5. The Ligand Scout software rapidly generated a 3D pharmacophore from the structural data of small molecules in a fully automated and appropriate way [34].

### 2.3. Library Preparation and Virtual Screening

About 20 libraries that contain 38,000 natural compounds (Flavonoids, Traditional Chinese Medicinal compounds, highly selective inhibitors, antiviral and bioactive compounds) were downloaded from the SelleckChem (https://www.selleckchem.com/screening-libraries.html (accessed on 2 April 2022)) database on the basis of the Rule of Five and minimizing their energy via UCSF Chimera 1.14 before docking to make sure they were in the right conformation so the docking results would be more realistic. The system was subjected to energy minimization to start the production runs. The system was minimized using the steepest decent [35] and conjugate algorithm [36]. A total of 1500 steps of conjugate gradient algorithm were applied on the system in which after every 50th step, the deepest descent algorithm was applied. The freely available UCSF Chimera (https://www.cgl.ucsf.edu/chimera/ (accessed on 8 April 2022)) was used for visualization and analysis of molecular structures together with density maps, trajectories and sequence alignments. The pharmacophore model was used for the virtual screening of 38,000 natural compounds via Ligand Scout and it selected compounds that had the best pharmacophoric features and hit scores.

### 2.4. Docking Calculation and Interaction

After virtual screening, the top-20 compounds were selected on the basis of their pharmacophore fit score. Molecular docking of these compounds was performed through PyRx which contained Open Bebel, Vina Wizard, Autodock vina and python interpreter so that it could automatically convert files into the required file format. Active site residues were predicted via cocrystal structure and the CASTp server. The interpretation of H-bonds, polar, pi-anion, pi-alkyl, pi-donor hydrogen bond and hydrophobic interactions among HE receptor and studied compounds were visualized through UCSF Chimera 1.14 and PyMOL. On the basis of visualization, a best-hit compound was selected.

### 2.5. Toxicity Analysis and Bioactivity Prediction

ADMET analysis, medicinal chemistry, lead-like and drug-like properties were predicted via freely available web tools, such as SwissADME (http://www.swissadme.ch/ (accessed on 22 April 2022)) [37], pkCSM (http://structure.bioc.cam.ac.uk/pkcsm (accessed on 23 April 2022)) [38], ProTox-II (https://tox-new.charite.de/protox_II/ (accessed on 19 January 2023)) [39] and OSIRIS Property Explorer [40]. These tools analyzes drugs and compounds to check whether the designed drug/compound is nontoxic for humans or not. In addition, bioavailability radar analysis was performed to check the drug likeness and bioavailability of the identified compound.

### 2.6. Lead Identification

Lead identification/optimization is an imperative step in drug design. All calculations (docking scores, interaction and ADMET analysis including MW, HBD, HBA, logP, PSA, rotatable bonds and rings) were achieved for the identification of a lead compound having the most suitable results by following the rules i.e., Ghose, Veber, Egan and rule of five (ROF). The compound with good interaction, best fit-score and binding affinity was selected as a potential inhibitor against HE surface glycoprotein.

### 2.7. Molecular Dynamic (MD) Simulations

The lead compound protein and reference complex were subjected to MD simulation to evaluate changes in the internal dynamics of the target protein. Amber tools were used to prepare input files while NAMD3 was utilized to conduct 100ns MD simulation with ff14SB and Gaff forcefields for protein (HE glycoprotein) and ligands (calceolarioside B and control), respectively. Both ligands and proteins were prepared via the Antechamber and Leap program of Amber tools while long-range electrostatic interactions were computed by the particle mesh Ewald method and short-range interactions, such as columbic and van der Waals interactions, were calculated with a cutoff of 10 Å. A specific number of Na^+^ and Cl^−^ counter ions and a TIP3P water box [41] of size 10 Å were introduced to imitate physiological salt concentration and to neutralize the whole system. A shake algorithm [42] was employed to constrain all bond lengths containing hydrogen bonds to heavy atoms while the particle mesh Ewald method [43] was utilized to calculate the long-range electrostatic interactions. The system was minimized using 10,000 steps and water equilibration was performed by using 10,000 steps. The temperature equilibrations were performed gradually at 200, 250, and 300 K temperatures for 5000 steps. After equilibration, the system was ready and prepared complexes were used to run MD simulation at a constant temperature of 310 K and 1 atm pressure. MD trajectories for both systems were analyzed to obtain RMSD, Rg, RMSF, energy decomposition, PCA, cross correlation and H-bond plot analysis.

### 2.8. Molecular Mechanics/Generalized Born Surface Area (MMGBSA) Analysis

Molecular Mechanics/Generalized Born Surface Area (MMGBSA) is the efficient force field technique to access binding free energy of a system (ligand-receptor) in kcal/mol [44]. Calculations were based on the last 300 frames and determined by using the following equations.

ΔG_bind_ = G_complex_ − G_protein_ − G_ligand_ (1)

ΔG_bind_ = ΔG_gas_ + ΔG_sol_ − TΔS (2)

ΔG_gas_ = Bond + Angle + Dihed + EEL + VDWAAL (3)

ΔG_sol_ = ΔEGB + ΔESURF (4)

ΔG_bind_ is the total binding free energy of the system (Equation (1)) which is calculated by employing Equation (2). TΔS is the change of conformational entropy on ligand binding at a given temperature. ΔG_gas_ is the total of bond, angle, dihydral, EEL (electrostatic component of the internal energy) and van der Waals energy (Equation (3)). Internal energy is associated with the vibration and rotation of single bond torsional angles. Solvation free energy (ΔG_sol_) is the combination of ΔEGB (polar component of the solvation energy) and ΔESURF (no polar component of the solvation energy) (Equation (4)).

## 3. Results

**Figure 1:**
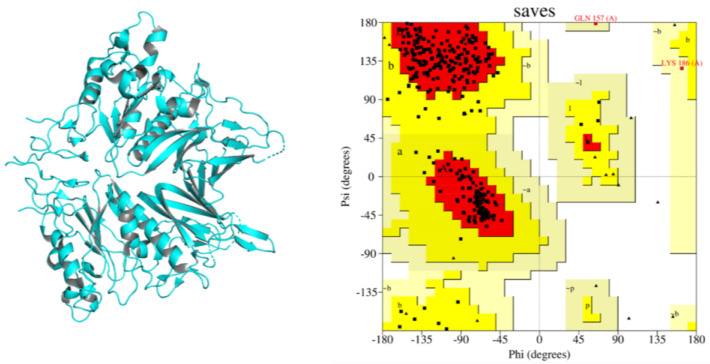
Structure of Hemagglutinin esterase surface glycoprotein (5N11) of human coronavirus (**left side**) along it’s Ramachandran Plot with different regions (Most favored, additional allowed regions and generously allowed regions) of targeted receptor (**right side**).

The target protein is the viral envelope protein, whose structure was retrieved from the Protein Data Bank with PDB ID 5N11 (Hemagglutinin esterase), 2.45 Å resolution and 0.249 Å R-free value. The HE receptor has two chains, 423 residues and 47,482 Da MW. The predicted structure was refined by removing the small compounds and water molecules via BIOVIA Discovery Studio, optimized, minimized and shown in Figure 1 along with the associated Ramachandran plot that provides information about the stereochemistry of the target protein. According to the graph, 87.2% residues are in most-favored regions, 12.1% are in additional allowed regions, while 0.7% are in generously allowed regions. Most of the residues are in most-favored regions and the overall quality of the HE protein is 95%, which indicates good quality structure.

According to the domain architecture of the HE protein, there are two domains, shown in Figure S1. The first one is hema_esterase (22–375) while the second one is Hema HEFG (129–262), which is required for infection by recognizing the host cell receptor and helping with the fusion of the viral and host cell membrane.

### 3.1. Ligand-Based Virtual Screening and Molecular Docking

**Figure 2:**
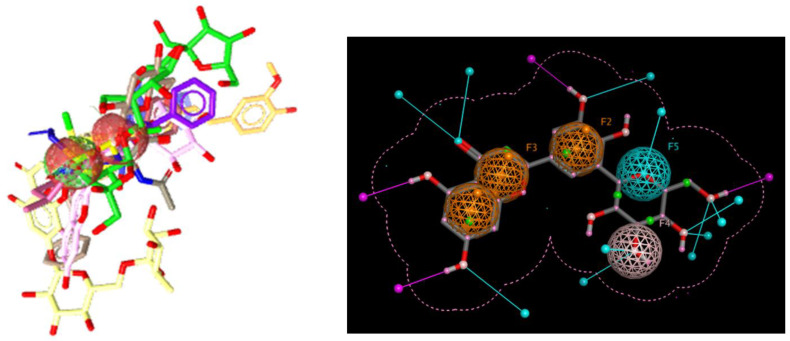
Ligands with merged pharmacophoric features at the left side and the final pharmacophore query at the right side.

**Figure 3:**
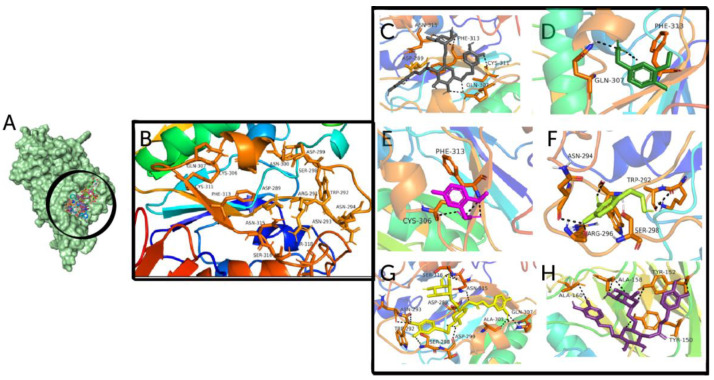
(**A**) Best docked poses of potential hits with HE glycoprotein (5N11) in the surface form. (**B**) Labelled binding site residues engaged in interaction. Ligands (**C**) Calceolarioside B (**D**) Homovanillic acid (**E**) 2-(5-fluoro-2-methoxyphenyl)acetic acid (**F**) Hydroxytyrosol (**G**) Omarigliptin (**H**) 4′-Methoxyresveratrol involved in the interaction with the receptor.

Fifteen antiviral compounds were used to generate the pharmacophore model which recognized the defined binding mode. The structures of these ligands along with their name and pharmacophore fit score are presented in Table 1. A total of 15 active compounds were aligned via Ligand Scout and we generated a pharmacophore model by choosing the best features, such as HBD, HBA and aromaticity. Ligands with merged and selected pharmacophoric features are shown in Figure 2. The prepared pharmacophore model was used to screen a library of 38,000 natural compounds. Virtual screening has become a standard tool in drug discovery. After virtual screening the top-20, hits with the best pharmacophore fit score were selected for molecular docking with 5N11 receptor to explore their binding modes. Figure 3A,B illustrates docked poses within the active site and residues engaged in the binding. Docking energies of the top-20 ligands are shown in Table 2. The natural compound Calceolarioside B illustrates 11 interactions and was found most favorable for HE inhibition with the least binding energy of −7.8 kcal/mol. Polar amino acid residues i.e., Gln307, Cys311, Asn315, Asp289, Asp299 and nonpolar Phe313 created a hydrogen bond with OH of Calceolarioside B to inhibit the activity of the HE protein (Figure 3C). The hydroxyl group (OH) increased the activity by participating in hydrogen bonding. The homovanillic-acid-HE complex formed two hydrogen bonds with OH of Phe313 while Gln307 formed hydrogen bond interactions with oxygen, respectively (Figure 3D). As shown in Figure 3E, 2-(5-fluoro-2-methoxyphenyl)acetic acid formed a network of 02 conventional hydrogen bond interactions with polar Cys306 residue, and aromatic residue Phe313 while non-polar aliphatic Ala303, aromatic Tyr312 and polar uncharged Gln349 make carbon hydrogen bonds. (Figure 3F) Hydroxytyrosol formed three conventional hydrogen bonds with Asn294, Trp292, and Arg296 while one carbon hydrogen bond with polar uncharged Ser298. Figure 3G depicts the hydrogen bond interactions of omarigliptin with Ser316, Arg291, Asn315, Ala303, Ser298, Asp299, Trp292, Asn293 and Gln307 residues. In addition, 4′-Methoxyresveratrol formed hydrogen bonds with Ala158, Tyr150, Tyr152 and Ala160 and pi–pi interactions with residue Ala173. The remaining 14 compounds displayed interactions with binding site residues as shown in Table S2.

**Figure 4:**
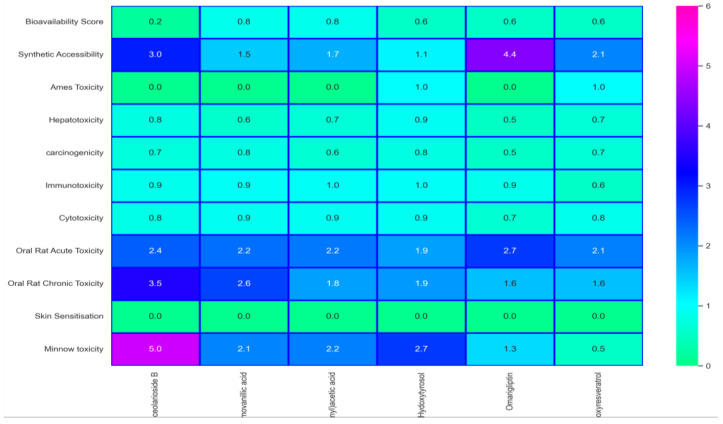
Toxicity analysis of the top-6 phytochemicals.

**Figure 5:**
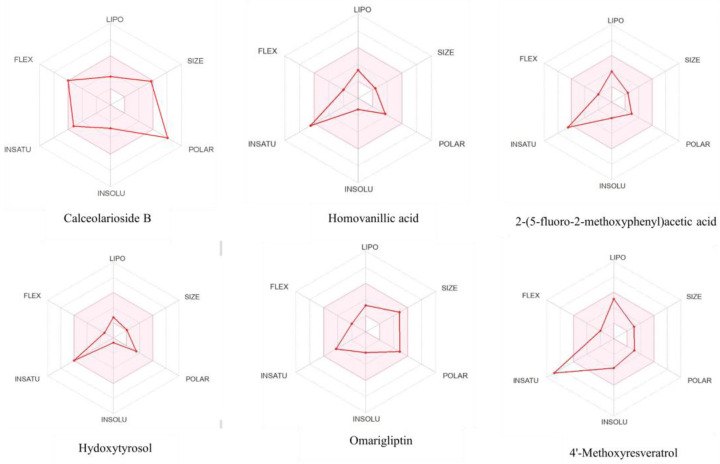
Bioavailability radar based on the physicochemical properties of top-6 hits.

ADMET properties and heatmap for toxicity analysis were determined for the top-six compounds in Table 3 and Figure 4, respectively, that lay within the acceptable toxicity profile. The bioavailability radar of the six top hits is shown in Figure 5. Phytochemical calceolarioside B was selected as a lead compound against HE glycoprotein of human coronavirus on the basis of its best binding affinity, RMSD, good interactions, pharmacophore fit score and ADMET properties.

### 3.2. Molecular Dynamics Simulations

To further analyze the stability of the lead compound-receptor and control complex, MD simulation was performed, and we evaluated MD trajectories and determined the RMSD, RMSF, Radius of Gyration, principal component analysis (PCA), cross correlation, no. of hydrogen bonds and MMGBSA.

#### 3.2.1. System Stability, Fluctuation and Radius of Gyration

**Figure 6:**
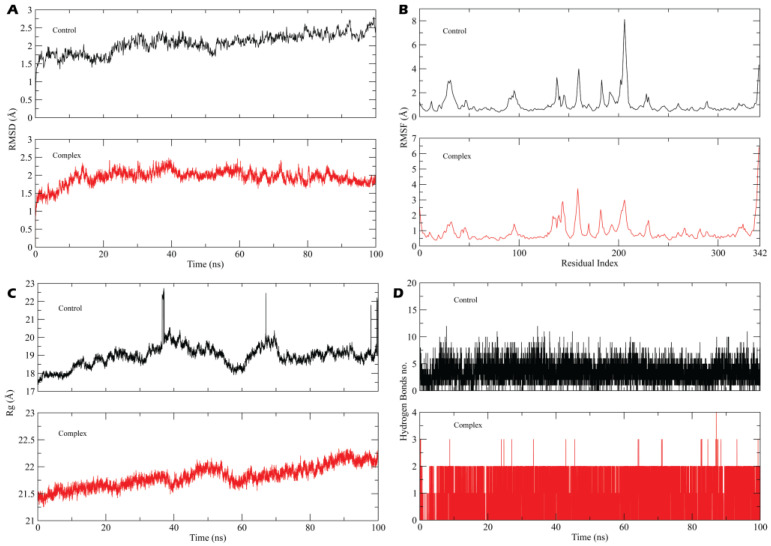
(**A**) RMSD plot of the control and calceolarioside B in contact with the target protein (complex) during 100,000 ps, (**B**) RMSF plot of both the control and complex systems which evaluates the structural flexibility of each residue, (**C**) Radius of Gyration (Rg) of the control and hit compound-HE system over 100 ns, (**D**) Hydrogen bond plot depicting all H-bonds formed between the lead-receptor and control system throughout the simulation time.

Figure 6 demonstrates RMSD, RMSF, Rg and hydrogen bond plot of control and calceolarioside B-HE complex for 100 ns. MD trajectory displayed an average RMSD value of 1.58 ± 0.10 Å for the complex and 2.0 ± 1.10 for the control while there was a slight increase in the RMSD of the control at 22 ns (Figure 6A). The RMSD value of the control and the complex did not vary significantly, staying nearly constant over the course of the simulation and represented rigidity (Figure 6B). To identify the dynamic behavior of most mobile residues and the effect of calceolarioside B binding on the flexibility of the target protein, RMSF was investigated. According to the RMSF plot, protein residues did not experience much flexibility when binding with a hit and reference compound but a noteworthy escalation in the flexibility of amino acids was analyzed between 165–205 residues while an escalation in flexibility was remarkable in the case of the control. Except for the specific region (165–205), both systems illustrated similarity in residual fluctuations as the average RMSF value of the complex was 1.10 ± 0.25 Å and 1.50 ± 0.30 Å for the control. The higher RMSF value at the C and N terminal and the middle residues represented loop regions that fluctuated more than other secondary structures while a lower RMSF represented relatively rigid residues. We detected the compactness of hemagglutinin esterase by approaching the radius of gyration (Rg) of carbon alpha atoms. It provided insights into the overall protein dimensions and enabled evaluations of the modifications to the tertiary structure of the protein throughout the simulation. Figure 6C displays a fluctuation near 40 and 58 ns in the complex system while the control represents significant fluctuations at 35, 68 and 98 ns with an abrupt decrease near 60 ns and a sustained average 20.50 Å Rg value. Overall, there was no major variation in the complex Rg throughout the simulation which showed that there were no unfolding events or loose packing, and revealed the extremely compacted nature of protein–ligand complex, and the complex maintained a 21.74 ± 0.18 Å Rg value.

(Figure 6D) The time evolution plot of H-bonds determines the formation and stability of hydrogen bonds throughout the simulation time as H-bonds play a significant role in drug specificity, metabolism and absorption [45,46]. The results illustrated that calceolarioside B formed up to four H-bonds with 88.34% occupancy and depicted the stable nature of the complex while the control formed up to ten hydrogen bonds with a minimum of six H-bonds throughout the simulation period.

#### 3.2.2. Principal Component Analysis (PCA)

**Figure 7:**
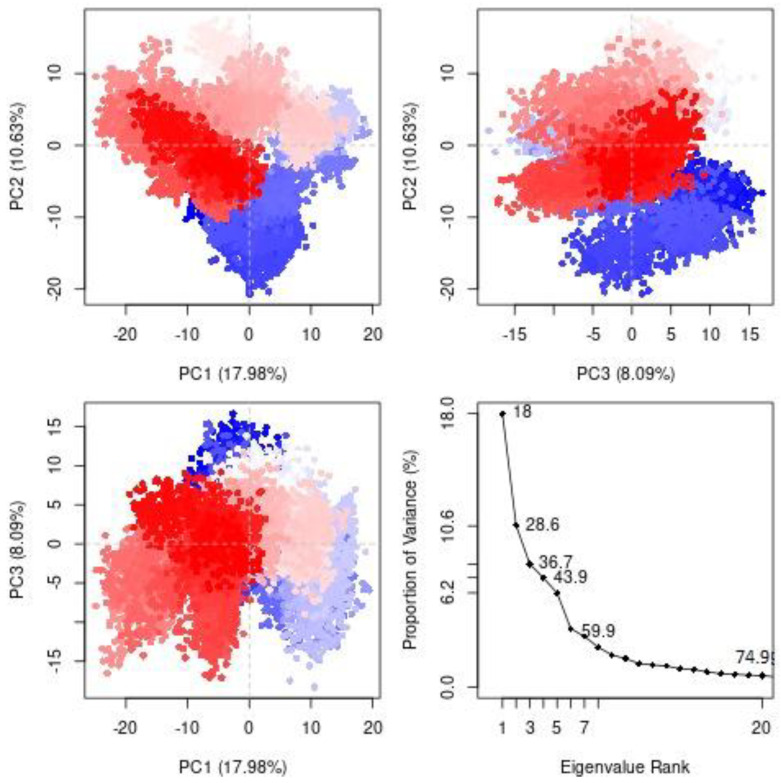
The interpretation of variance (HE-calceolarioside B) against eigenvalues calculated by Principal Component Analysis. The 3 PCs showed fluctuating regions with 36.7% overall fluctuations. The fluctuations in PC1, PC2 and PC3 were 17.98%, 10.63% and 8.09%, respectively.

A Principal Component Analysis (PCA) was employed to detect the protein’s conformational changes mediated by calceolarioside B binding and reveal the collective motions of MD trajectories. According to Figure 7, PC1, PC2, PC3 and eigenvalues of receptor was plotted against the respective eigenvector index for the first 20 modes of motion. PC analysis indicated conformational changes in all clusters where the blue region exhibited the most significant movements, the white region represented intermediate movements and the red region displayed the least flexible movements. Overall protein movement was controlled by eigenvectors, especially the higher ones and the top-five eigenvectors in our system demonstrated dominant movements with eigenvalues of 18.0–59.9% while the remaining eigenvectors had lower eigenvalues. According to the PCA plot, the PC1 cluster retained the highest variability of 17.98%, PC2 illustrated 10.63% variability, while PC3 showed minimal variability (8.09%). The minimal variability of PC3 indicates highly stabilized protein ligand binding and a compact structure when compared to the PC1 and PC2 clusters.

#### 3.2.3. Positive-Negative Correlation Movements of Residues

**Figure 8:**
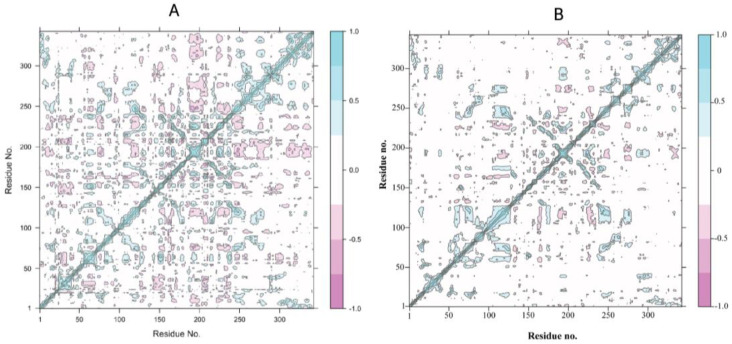
Dynamic cross-correlation matrix of Cα atoms of (**A**) the control and (**B**) target-protein-bound calceolarioside B. The cyan color depicts a high correlation while the magenta color designates anticorrelation between amino acid residues.

Dynamic cross-correlation maps represent inter residual motions computed via MD trajectories (Figure 8). The cyan and magenta color depicts strongly correlated (positive) and anticorrelated (negative) motions, respectively, between essential residues throughout the MD simulation. The correlated residues were more than 0.8 while anticorrelated residues were <−0.4. Positive correlation confirms RMSD and revealed a high stability. As shown in Figure 8A, the control (standard compound-HE) depicts a positive correlation and also depicts some of the negatively correlated movements (Figure 8B). The lead compound and receptor are significantly correlated, and positively correlated movements were extremely notable at residues 100–130 and 250–350 (active site region) and a higher percentage of pairwise-correlated residues represent the stable binding of calceolarioside B with the HE protein.

#### 3.2.4. Binding Energy Landscape and Energy Decomposition Analysis

**Figure 9:**
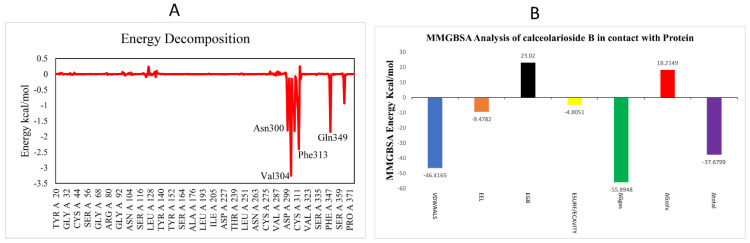
(**A**) Per-residue energy decomposition analysis (**B**) MMGBSA-based binding free energy of calceolarioside B-HE system in kcal/mol.

To estimate the contribution of individual residues towards HE protein’s inhibition, MMGBSA and energy decomposition analyses were performed. Per-residue energy decomposition analysis showed the contribution of different amino acids to the overall binding energy. According to the energy decomposition graph, the highest contributing residues Val304, Phe313, Gln349 and Asn300 with −3.3, −2.5, −2, −1.5 kcal/mol energies interacted with the ligand and are highlighted in Figure 9A.

Highly dynamic, cost-effective and computer-derived MMGBSA analysis computes the binding free energy of the protein–ligand complex at the molecular level that might be extremely beneficial for drug design (Figure 9B). The binding free energy (ΔG_bind_) of calceolarioside B complex with HE protein is −37.6799 kcal/mol. Data reveal that van der Waals interactions (VDWAALS) significantly contribute (−46.4165 kcal/mol) to the binding free energies while EGB was 23.0200 kcal/mol and EEL was −9.4782 kcal/mol. The ΔG_gas_ (bond + angle + dihed + EEL + VDWAALS) was the highest energy with −55.8948 kcal/mol value (Table S1). The Calceolarioside B-HE complex represented the lowest negative values, indicating stability and favorable binding of calceolarioside B in the active site of the HE receptor.

## 4. Discussion

The pandemic outbreak of novel human coronavirus spread into several other countries. In 2020, WHO declared a global health emergency based on the growing number of cases and a situation that was growing worse on a daily basis. To handle this situation, it is necessary to develop new drugs to treat COVID-19. Therefore, we used a multidisciplinary field, computer-aided drug design widely used to find new drug candidates in less time and at a reduced cost.

In this research, we made use of different bioinformatics tools to find a natural inhibitor against the HE surface glycoprotein of human coronavirus. In recent years, the use of natural compounds against viral infections has been found to be effective and is gaining importance. Natural compounds are less toxic, and less harmful to human health and are being analyzed to understand whether they could inhibit coronavirus. This virus shares sequence similarity with beta coronaviruses which possess the HE protein that interacts with various types of sialic acid, removes acetyl groups from O-acetylated sialic acid and play a role in binding to the target cell.

Ligands and receptor were obtained from PubChem and PDB respectively, then minimize their energy in order to reduce the overall potential of the receptor and ligands and to make sure that they were in the right conformation with low delta G values so as to be considered close to the biological system. Libraries of natural inhibitor compounds were downloaded from the SelleckChem database which provided the antiviral, antifungal and anti-inflammatory effects. A pharmacophore model was generated on the basis of shared steric and electronic features of all known active and antiviral compounds with a wide range of structural diversity and activities were aligned which were responsible for the biological interactions. The pharmacophore model explains how structurally distinct ligands can bind to the same side of the receptor.

Virtual screenings of thousands of natural compounds have been performed to discover novel molecules from a library of 38,000 compounds, docked via PyRx and to determine the interaction between the small molecule and the active site of the target protein at an atomic level. Resultantly, we selected the best compound, calceolarioside B, on the basis of its best docking affinity, RMSD and other physiochemical properties. The lead compound tightly bound with Asn315, Val304, Phe313, Asp289, Cys311, Asn300 and Gln307 amino acids and stabilized the active site of the HE receptor. Calceolarioside B is derived from the roots and leaves of *Fraxinus sieboldiana Blume* plant, which is member of the Oleaceae family, commonly known as the ash tree that is found in various regions of the world. It is native to China Southeast, Japan and Korea. In northern areas of Pakistan, *Fraxinus sieboldiana Blume* plant is usually used to treat malaria and pneumonia. Metabolites and extracts from this plant exhibit a wide range of biological actions, including anticancer, anti-inflammatory, antioxidant, antimicrobial, hepatoprotective, antiallergic and anti-viral properties.

A toxicity analysis evaluated the safety of the potential drug candidate, which indicated that calceolarioside B is safe to use in the future against the HE glycoprotein of human coronavirus. In checking for toxicity, the Lipinski rule was required to be followed to check whether the drug was toxic or non-toxic. The Calceolarioside B compound violated two Lipinski rules, but a drug or compound with two violations is acceptable while more than two violations are not acceptable. RMSD, which computes the average distance and the binding of calceolarioside B, revealed stability in the HE receptor. The RMSF graph did not represent major fluctuations in the target protein after binding of a hit compound. The radius of gyration value represents the compactness and stabilized folding in the phytochemical bound complex. Calceolarioside B formed a lot of interactions with the HE receptor, but the H-bond played a significant role by stabilizing the complex. PCA, DCCR and MMGBSA represent compactness and stability in the lead compound-HE receptor. Generally, the analysis of the MD simulation trajectory revealed the stable and energetically favorable complex formation in the presence of a lead phytochemical. These findings and their implications are discussed in the broadest context possible. This study will help researchers to evaluate the compounds that are effective and beneficial against human coronavirus.

## 5. Conclusions

HE glycoprotein (5N11) is involved in causing COVID-19 disease in humans. This research work was designed to find a natural compound that can act as inhibitor against HE glycoprotein of human coronavirus within a reduced time and cost by using different bioinformatics tools. Pharmacophore modeling, virtual screening and molecular docking helped to filter out calceolarioside B as having a low binding affinity with the target protein. *Fraxinus sieboldiana Blume*, a medicinal plant with rich phytochemical compounds, out of which calceolarioside B is one of the compounds that show antiviral activity, inhibits the replication of coronavirus, stabilizing the structure and energy of the HE receptor indicated via MD simulation and MMGBSA analysis. The selected lead phytochemical must be validated in future through in vitro and in vivo studies. It is concluded that Calceolarioside B that is present in the root bark and leaves of the *Fraxinus sieboldiana Blume* plant, is an effective lead compound in the case of novel coronavirus.

## Figures and Tables

**Table 1:** Chemical structures and their names, along with the pharmacophore fit score of active compounds.

No.	Names	Pharmacophore Fit Score	Structures
1	5-Norbornene 2,3 dicarboxy-chloride	32.99	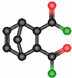
2	Levan	34.31	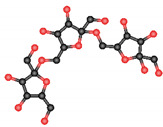
3	Caffeic Acid	40.44	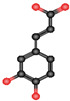
4	S-Nitroso-N-Acetylpenicillamine	41.00	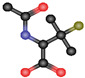
5	Curcumin	41.75	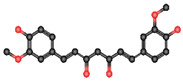
6	Quercetin	41.60	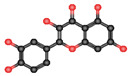
7	Diallyl disulphide	33.22	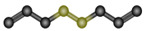
8	Pulegone	37.29	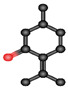
9	Flavylium	13.27	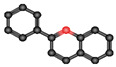
10	Pinocembrin	34.61	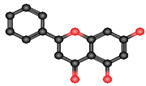
11	Gallic acid	34.68	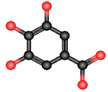
12	Rosmeric acid	41.50	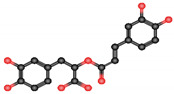
13	Luteolin	34.67	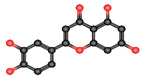
14	Hesperidin	41.61	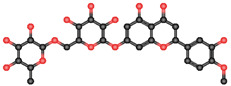

**Table 2:** Binding affinities, pharmacophore fit score and physicochemical properties of hit compounds.

Selected Compounds	Docking Energies	Pharmacophore Fit Score	HBA	HBD	Rotatable Bonds	M.W	logP
Calceolarioside B	−7.8	40.08	10	7	9	478.4	0.6
Homovanillic acid	−7.7	40.05	4	2	3	182	0.4
2-(5-fluoro-2-methoxyphenyl)acetic acid	−7.7	40.00	4	1	3	184	1.5
Hydroxytyrosol	−7.7	40.05	3	3	2	154.16	0.17
Omarigliptin	−7.7	39.86	8	1	3	398.4	0.3
4′-Methoxyresveratrol	−7.7	39.86	3	2	3	242.27	3.5
12-Hydroxy-10,13-dimethyl-2,4,5,6,17-dione	−7.7	39.96	8	2	3	391.4	0.3
AZ628	−7.6	40.4	5	2	5	451.5	4.2
Telaprevir	−7.5	39.99	8	4	14	679.8	4.2
Verdinexor	−7.5	39.87	11	2	5	422.3	4.1
4-[3-(morpholine-4-carbonyl)-5-[4-(trifluoromethyl)phenyl]pyrazol-1-yl]benzenesulfonamide	−7.5	39.93	9	1	4	480.5	2.4
3,4 dihydroxyphenylacetic acid	−7.3	40.08	4	3	2	168.15	0.5
aminomethyl(phenyl)phosphinic acid	−7.3	40.00	3	2	2	171.13	−2.7
3-[2-(3-cyanatophenoxy)ethoxy]phenyl]cyanate	−7.0	39.89	6	0	7	296.28	3.9
N-[(4,5-difluoro-1H-benzimidazol-2-yl)methyl]-9-(3-fluorophenyl)-2-morpholin-4-ylpurin-6-amine	−6.5	39.87	10	2	5	480.4	3.5
N-(2-methyl-4-phenylbut-3-en-2-yl)-1-phenylmethanimine	−6.4	40.4	1	0	4	249.3	4.4
Ruboxistaurin	−6.2	40.4	4	1	2	468.5	2.7
Daunorubicin	−5.3	40.11	11	5	4	527.5	1.8
Forsythoside A	−5.1	40.08	15	9	11	624.6	−0.5
Turofexorate Isopropyl	−5.1	40.4	5	1	4	438.5	5.0

**Table 3:** ADMET analysis of the top-6 compounds.

Phytochemicals	Calceolarioside B	Homovanillic Acid	2-(5-fluoro-2-methoxyphenyl)acetic Acid	Hydroxytyrosol	Omarigliptin	4′-Methoxyresveratrol
Formula	C_23_H_26_O_11_	C_9_H_10_O_4_	C_9_H_9_FO_3_	C_8_H_10_O_3_	C_17_H_20_F_2_N_4_O_3_S	C_15_H_14_O_3_
Pfizer Rule	Accepted	Accepted	Accepted	Accepted	Accepted	Rejected
Golden Triangle	Accepted	Rejected	Rejected	Rejected	Accepted	Accepted
BBB Penetration	BBB+	BBB+	BBB+	BBB+	BBB+	BBB+
Fu	5.8%	18.71%	5.98%	61.31%	73.074%	1.403%
Density	1.048	1.01	1.037	0.982	1.118	0.935
ESOL Class	Soluble	Very soluble	Soluble	Very soluble	Soluble	Soluble
Ali Class	Moderately soluble	Very soluble	Soluble	Very soluble	Very soluble	Moderately soluble
Silicos-IT class	Soluble	Soluble	Soluble	Soluble	Soluble	Soluble
GI absorption	Low	High	High	High	High	High
Pgp substrate	Yes	No	No	No	Yes	No
log Kp (skin permeation)	−8.80	−7.18	−6.39	−7.75	−8.55	−5.33
Lipinski violations	2	0	0	0	0	0
Ghose violations	1	0	0	1	0	0
Veber violations	1	0	0	0	0	0
Acute Toxicity Alert	0	0	0	0	0	0
Genotoxic Carcinogenicity Alerts	1	0	0	0	0	0
SureChEMBL Rule Alert	0	0	0	0	0	0
Synthetic Accessibility	2.96	1.49	1.71	1.08	4.40	2.08
Drug-likeness	−0.05	0.17	−2.0	−1.3	3.65	−3.1
Drug Score	0.56	0.75	0.54	0.59	0.85	0.27
Mutagenicity	No	No	No	No	No	No
Tumorgenic	No	No	No	No	No	No
Irritant	No	No	No	No	No	No
Reproductive Effect	No	No	No	No	No	Yes

## Data Availability

Additional data are available upon request.

## Supplementary Materials

The following supporting information can be downloaded at: https://www.mdpi.com/article/10.3390/biomedicines11030793/s1. Figure S1: Domain architecture of HE Protein. Table S1. Calculated values of MMGBSA with different parameters of HE protein- calceolarioside B complex. Table S2. (A) 12-Hydroxy-10,13-dimethyl-2,4,5,6,17-dione, (B) AZ628, (C) Telaprevir, (D) Verdinexor, (E) 4-[3-(morpholine-4-carbonyl)-5-[4-(trifluoromethyl)phenyl]pyrazol-1-yl]benzenesulfonamide, (F) 3,4 dihydroxyphenylacetic acid (G) aminome-thyl(phenyl)phosphinic acid, (H) 3-[2-(3-cyanatophenoxy)ethoxy]phenyl] cyanate, (I) N-[(4,5-difluoro-1H-benzimidazol-2-yl)methyl]-9-(3-fluorophenyl)-2-morpholin-4-ylpurin-6-amine, (J) N-(2-methyl-4-phenylbut-3-en-2-yl)-1-phenylmethanimine, (K) Ruboxistaurin, (L) Daunorubicin, (M) Forsythoside A (N) Turofexorate Isopropyl.
