# Supplementary material for: Identification of Natural Lead Compounds against Hemagglutinin-Esterase Surface Glycoprotein in Human Coronaviruses Investigated via MD Simulation, Principal Component Analysis, Cross-Correlation, H-Bond Plot and MMGBSA
Source: Biomedicines. 2023 Mar 6;11(3):793. doi: 10.3390/biomedicines11030793 (PMC10044901; doi:10.3390/biomedicines11030793)
Supplement: Supplementary file 1 [file biomedicines-11-00793-s001.zip › biomedicines-2166501-supplementary.pdf]

Supplementary Materials

Identification of Natural Lead Compounds against Hemagglutinin Esterase Surface Glycoprotein in Human Coronaviruses Investigated via MD Simulation, Principal Component Analysis, Cross-Correlation, H-bond Plot and Mmgbsa

Iqra Ali, Muhammad Asif Rasheed, Simona Cavalu , Kashif Rahim, Sana Ijaz, Galal Yahya, Lucky Poh Wah Goh and Mihaela Popoviciu

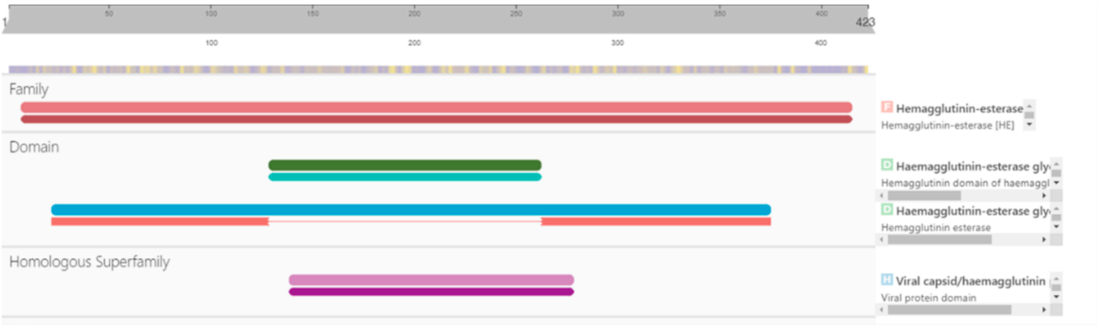

Figure S1. Domain architecture of HE Protein.

Table S1. Calculated values of MMGBSA with different parameters of HE protein- calceolarioside B complex.

Binding Energy Component	Average	Std. Dev.	Std. Err. of Mean
VDWAALS	-46.4165	3.6572	0.2111
EEL	-9.4782	3.4782	0.2008
EGB	23.0200	3.0008	0.1733
ESURF	-4.8051	0.3681	0.0213
DELTA G gas	-55.8948	5.6366	0.3254
DELTA G solv	18.2149	2.8397	0.1639
DELTA TOTAL	-37.6799	3.7919	0.2189

Table S2. (A) 12-Hydroxy-10,13-dimethyl-2,4,5,6,17-dione, (B) AZ628, (C) Telaprevir, (D) Verdinexor, (E) 4-[3-(morpholine-4-carbonyl)-5-[4-(trifluoromethyl)phenyl]pyrazol-1-yl]benzenesulfonamide, (F) 3,4 dihydroxyphenylacetic acid (G) aminomethyl(phenyl)phosphinic acid, (H) 3-[2-(3-cyanatophenoxy)ethoxy]phenyl] cyanate, (I) N-[(4,5-difluoro-1H-benzimidazol-2-yl)methyl]-9-(3-fluorophenyl)-2-morpholin-4-ylpurin-6-amine, (J) N-(2-methyl-4-phenylbut-3-en-2-yl)-1-phenylmethanimine, (K) Ruboxistaurin, (L) Daunorubicin, (M) Forsythoside A (N) Turofexorate Isopropyl.

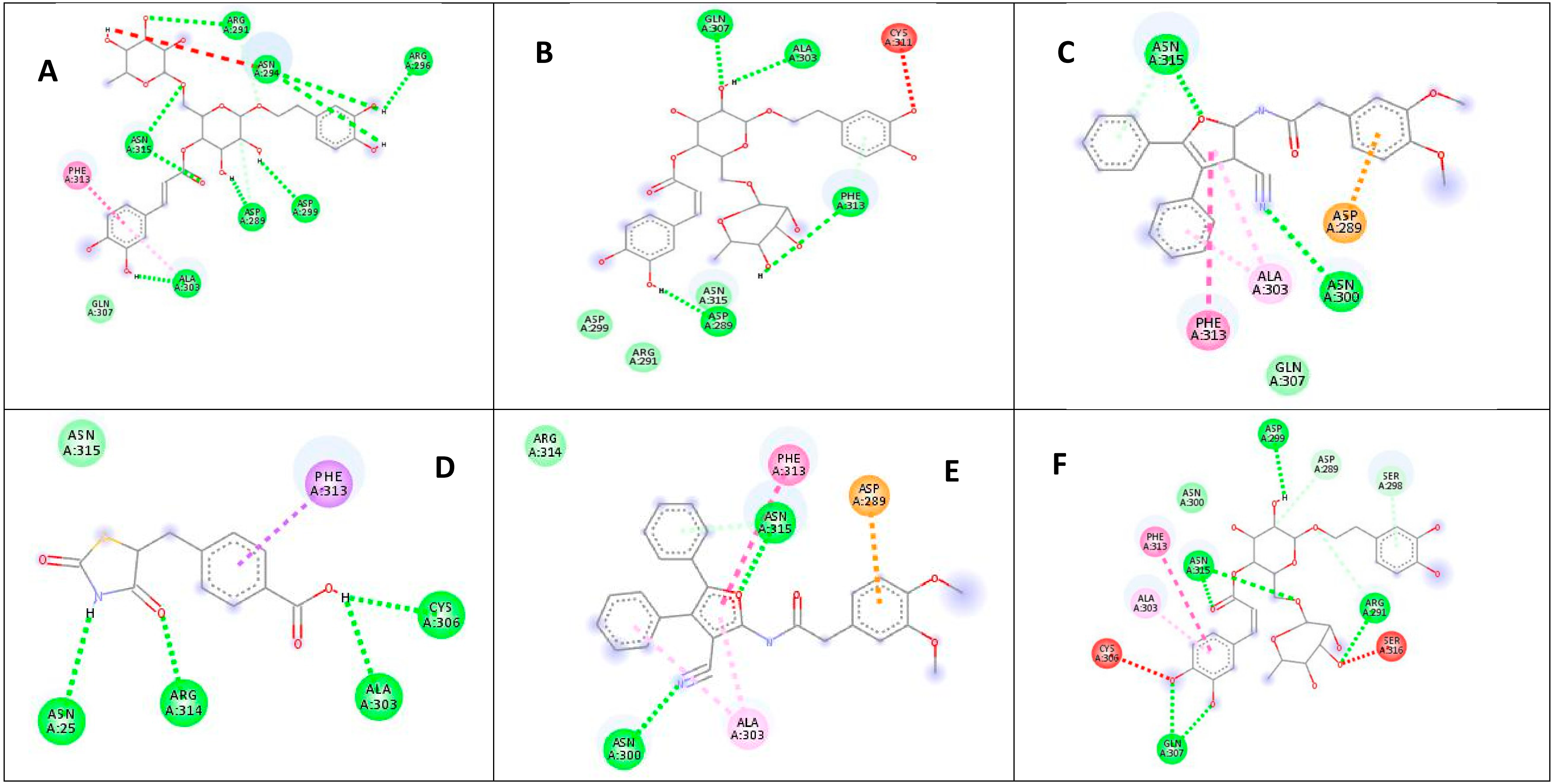
A
ARG A:291
ASN A:294
ARG A:296
ASN A:315
PHE A:313
ASP A:289
ASP A:299
ALA A:303
GLN A:307
B
GLN A:307
ALA A:303
CYS A:311
PHE A:313
ASN A:315
ASP A:289
ASP A:299
ARG A:291
C
ASN A:315
ASP A:289
ALA A:303
ASN A:300
PHE A:313
GLN A:307
D
ASN A:315
PHE A:313
CYS A:306
ASN A:25
ARG A:314
ALA A:303
E
ARG A:314
PHE A:313
ASP A:289
ASN A:315
ASN A:300
ALA A:303
F
ASP A:299
ASP A:289
SER A:298
ASN A:300
PHE A:313
ASN A:315
ALA A:303
ARG A:291
CYS A:306
SER A:316
GLN A:307

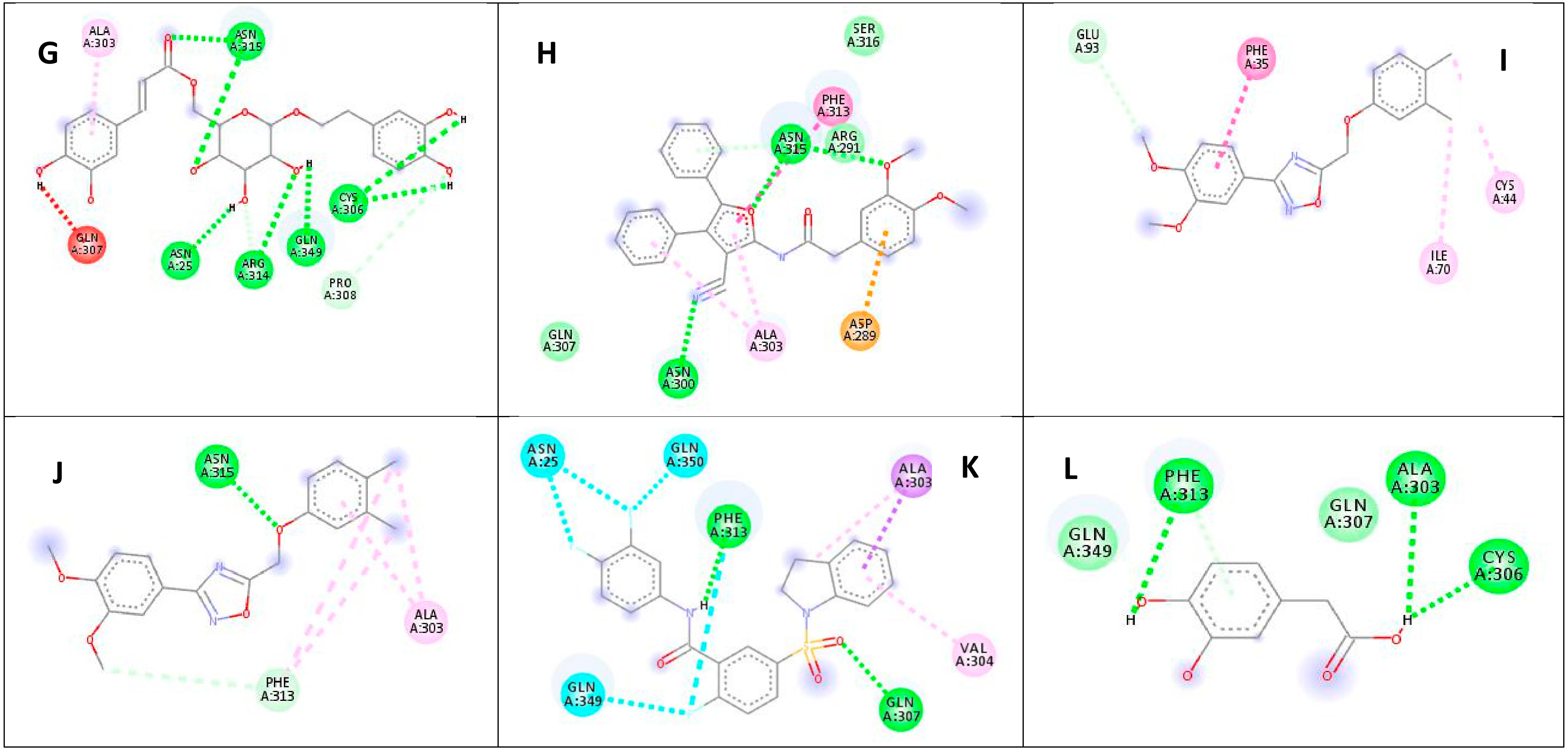
G
ALA A:303
ASN A:315
CYS A:306
GLN A:307
ASN A:25
ARG A:314
GLN A:349
PRO A:308
H
SER A:316
PHE A:313
ASN A:315
ARG A:291
GLN A:307
ALA A:303
ASP A:289
ASN A:300
I
GLU A:93
PHE A:35
CYS A:44
ILE A:70
J
ASN A:315
ALA A:303
PHE A:313
K
ASN A:25
GLN A:350
PHE A:313
ALA A:303
VAL A:304
GLN A:349
GLN A:307
L
PHE A:313
GLN A:349
GLN A:307
ALA A:303
CYS A:306
